# A systematic review and meta-analysis of the long-term effects of physical activity interventions on objectively measured outcomes

**DOI:** 10.1186/s12889-023-16541-7

**Published:** 2023-09-02

**Authors:** J. Gasana, T. O’Keeffe, T. M. Withers, C. J. Greaves

**Affiliations:** 1 School of Health Sciences, College of Medicine and Health Sciences, University of Rwanda, P.O Box 3286, Kigali, Rwanda; 2 School of Sport, Exercise and Rehabilitation Sciences, University of Birmingham, Birmingham, B15 2TT UK

**Keywords:** Physical activity intervention, Objective outcome measure, Systematic review, Adults, Randomised control trials

## Abstract

**Background:**

Although physical activity interventions are frequently reported to be effective, long-term changes are needed to generate meaningful health benefits. There are criticisms that evaluations of physical activity interventions mostly report short-term outcomes and that these are often self-reported rather than measured objectively. This study therefore aimed to assess the long-term (at least 24 month) effectiveness of behavioural interventions on objectively measured physical activity.

**Methods:**

We conducted a systematic review with a meta-analysis of effects on objectively measured physical activity. We searched: Cochrane CENTRAL, EMBASE, PsychInfo, CINAHL and Pubmed up to 10th January 2022. Studies were included if they were in English and included a physical intervention that assessed physical activity in the long-term (defined as at least 24 months).

**Results:**

*Eight* studies with 8480 participants were identified with data suitable for meta-analysis. There was a significant effect of interventions on daily steps 24 months post baseline (four studies, SMD: 0.15, 95% CI: 0.02 to 0.28) with similar results at 36 to 48 months of follow up (four studies, SMD: 0.17, 95% CI: 0.07 to 0.27). There was a significant effect of interventions on moderate-to-vigorous physical activity 24 months post baseline (four studies, SMD: 0.18 95% CI: 0.07 to 0.29) and at 36 to 48 months (three studies, SMD: 0.16 95% CI: 0.09 to 0.23). The mean effect size was small. However, the changes in moderate-to-vigorous physical activity and steps per day were clinically meaningful in the best-performing studies.

**Conclusion:**

This review suggests that behavioural interventions can be effective in promoting small, but clinically meaningful increases in objectively measured physical activity for up to 48 months. There is therefore a need to develop interventions that can achieve greater increases in long-term physical activity with greater efficiency.

**Supplementary Information:**

The online version contains supplementary material available at 10.1186/s12889-023-16541-7.

## Background

Regular physical activity is essential for health and helps to prevent and treat non-communicable diseases such as hypertension and type 2 diabetes, as well as improving mental health and quality of life in all age groups [1, 2]. Moreover, being active reduces falls and increases quality of life in older people [3].

However, despite national and international guideline recommendations to engage in at least 150 min per week of moderate to vigorous level of physical activity [3–6], at least 28% of adults worldwide do not achieve this target, with women (32%) being less active than men (23%). Time spent doing moderate to vigorous physical activity also declines dramatically with increased age, with 65% of older adults being less active than recommended [7].

Crucially, in most cases, long-term changes in physical activity are needed to generate meaningful health benefits. Short-term changes may be beneficial in some contexts (e.g. pre-habilitation exercise prior to surgery [8], exercise to help alleviate a bout of depression [9], or to help manage nicotine cravings when stopping smoking [10]. However, many of the most important health benefits of physical activity, including reduced incidence of cardiovascular disease, cancers, type 2 diabetes and other chronic illnesses only accrue from extended engagement in physical activity over a number of years.

The effectiveness of interventions to increase physical activity has been reported in numerous studies involving a range of delivery modes, including face to face counselling, group based intervention, internet-based programmes and delivered in both primary care and the community [11, 12]. However, the existing evidence is limited mainly to changes in physical activity in the short to medium term (up to 12 months) [12].

A number of systematic reviews examining physical activity change have included studies that used subjective measures such as questionnaires and self-report diaries to measure physical activity [11, 13]. Although these are commonly used methods for measuring physical activity, their reliability has been questioned, as individuals’ recall of volume or intensity of physical activity tends to be imprecise [14]. In comparison, objective measurements, such as steps and minutes of moderate to vigorous physical activity assessed using electronic activity monitors (accelerometers or pedometers), may provide more precise measures of physical activity levels [15, 16]. A recent systematic review of interventions to increase physical activity in adults who were overweight or obese concluded that there was insufficient evidence on physical activity measured objectively beyond two years and that more studies reporting standardised objective measures for physical activity effectiveness at long term follow-up were needed [17]. A previous systematic review has reported on objectively measured physical activity include step counting measures only [18]. However, the data was analysed across all time points, so it is not possible to draw conclusions on long term effectiveness. In addition to this, the systematic review was unable to identify what components resulted in a successful intervention. A different systematic review [19] reported the effectiveness of maintenance interventions on device-measured physical activity reporting an overall standardised effect size of 0.14, equivalent to a 45 min per week increase in moderate to vigorous physical activity. However, the minimum follow-up was three months so it is not possible to draw conclusion on long term effectiveness. We therefore aimed to systematically review evidence on the long-term effects of physical activity in community-dwelling adults using objective measures. We also aimed to identify intervention characteristics associated with longer-term effectiveness.

## Methods

This systematic review and meta-analysis followed guidance from the Cochrane Collaboration [20]. The study was registered in the PROSPERO database of systematic reviews (CRD42019124377). As there is no universally agreed definition of long-term changes in physical activity [21], we defined long term as at least 24 months.

### Search strategy

The following electronic databases were searched for studies published up to 10th January 2022: the Cochrane Library CENTRAL (the Cochrane Central Register of Controlled Trials), Medline, Embase, CINAHL (Cumulative Index to Nursing & Allied Health Literature) and PsycINFO. The reference lists of included studies were also scanned for potentially relevant publications. An example search strategy is shown in Additional file 1. Only studies written in English are included in this study as there was no funding available for translation. Duplicates were removed using the duplicate removal function in EndNote (Clarivate Analytics, Philadelphia, Pennsylvania).

### Eligibility criteria

Articles were included in the systematic review if they met the following inclusion and exclusion criteria.

#### Inclusion

- The study was an individual or cluster randomised controlled trial.
- Participants were aged ≥ 18 years and above. This was a deviation from the registered protocol to ensure that interventions that are delivered through schools were excluded (as these interventions warrant a separate review).
- The intervention arm promoted lifestyle-based physical activity, including the promotion of physical activity to treat or prevent chronic diseases.
- The control group received no treatment, minimal intervention, or usual care.
- Physical activity was measured objectively at least 24 months post baseline. Credible metabolic indicators of the amount of physical activity undertaken, such as $\dot{\text{V}}$O_2peak_ (a measure of cardiorespiratory fitness) were also included [22].

#### Exclusion criteria

- Studies of structured exercise programmes designed to assess the effects of supervised exercise on metabolic outcomes (as opposed to promoting ongoing lifestyle physical activity or exercise).
- Participants living in a care home or supported accommodation.
- Physical activity was only measured subjectively (e.g. through self-report questionnaires, or exercise diaries).

### Data collection and analysis

#### Selection of studies

Two independent reviewers (JG, TOK or TW) screened the titles and abstracts to exclude studies that did not meet the inclusion criteria. The full texts of selected articles were also examined independently by reviewers (JG, TOK or TW) and in case of disagreement a fourth author (CG) was consulted to ensure agreement.

#### Data extraction and management

Two reviewers (JG, TOK or TW) independently extracted data from each included study using a data extraction tool. Data collected included study design, the country where the intervention took place, participants’ baseline characteristics including mean age, gender, BMI, ethnicity, setting, type and duration of the intervention, comparators and follow-up time points, along with any health conditions the participants had. The data extracted on physical activity outcomes were means and standard deviations of steps per day or per week and time spent in moderate-vigorous physical activity. Where studies reported a physical activity outcome at more than one time point, data were collected at all time points.

#### Assessment of risk of bias in included studies

Two reviewers (JG, TOK or TW) independently assessed risk of bias of the included studies using the revised Cochrane risk of bias tool for randomised trial RoB 2 [23]. The following domains were assessed: bias that could arise from the randomisation process (domain 1), bias due to deviations from the intended interventions (domain 2), bias due to missing outcome data (domain 3), bias due to outcome measurement (domain 4) and bias due to selective reporting of results (domain 5). However, due to the nature of the interventions studied (and the impossibility of blinding participants to group allocation), questions regarding double-blinding (specifically, questions 2.a and 2.b of domain 2) were not incorporated in the quality assessment process.

#### Synthesis of results /statistical analyses

Meta-analyses were performed using Review Manager software version 5.3 (RevMan 5.3). Since different measures were used to assess physical activity and interventions varied substantially, standardised mean difference (SMD) and random effects were used. Heterogeneity was assessed using the I^2^ statistic. Separate meta-analyses were conducted for minutes of moderate to vigorous physical activity per week and steps per day. For each analysis, studies were grouped into two time-points: 24 months from baseline and > 24 months (longer-term follow-up ranged from 36 to 48 months). Where the study involved more than one intervention arm, we extracted data for each arm of the study and the intervention arms were compared to the same control group separately. The results of studies that did not provide data suitable for meta-analysis were described narratively.

## Results

### Study selection

**Fig. 1:**
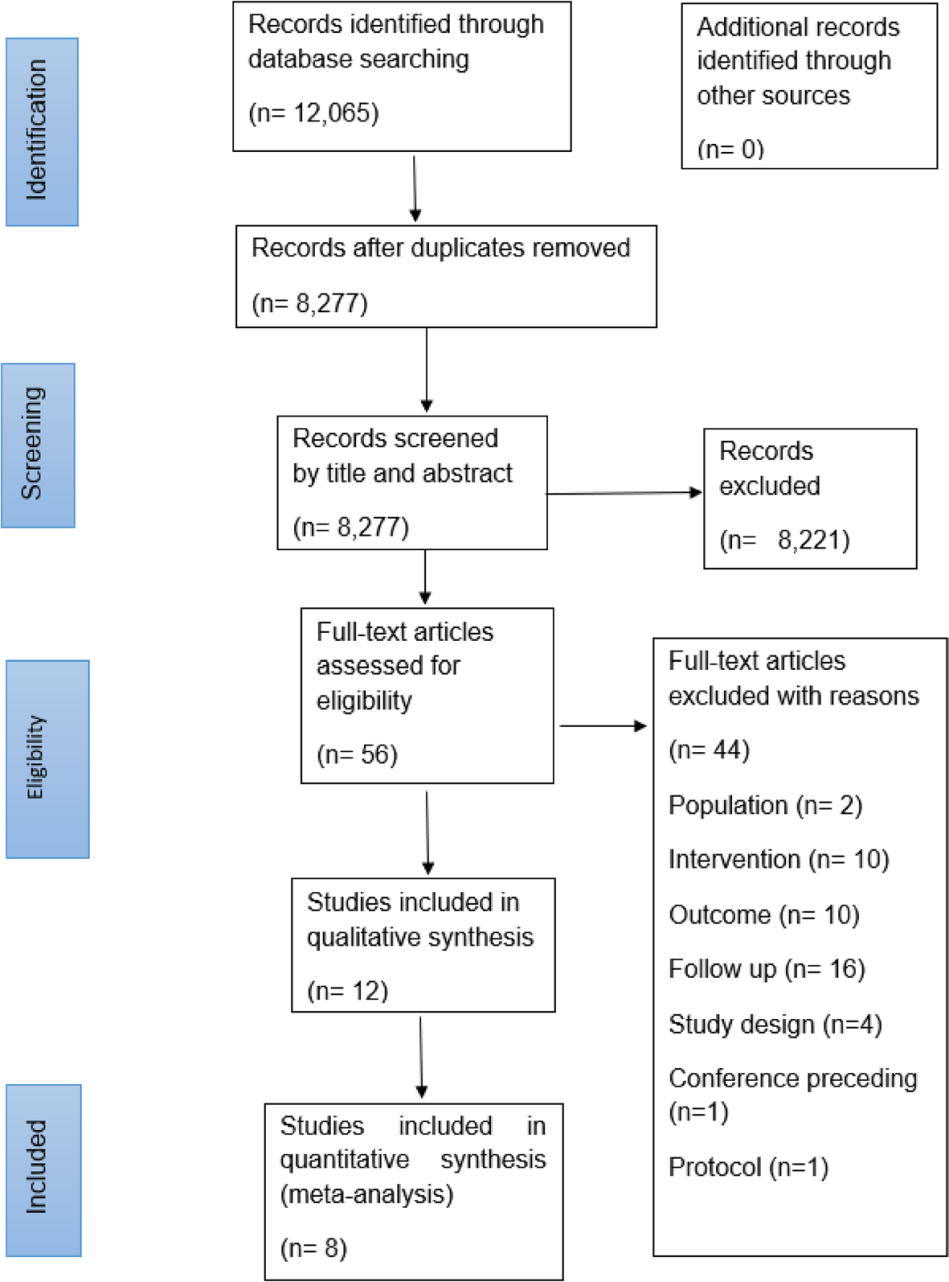
Prisma flow diagram of included studies

Figure 1 provides an overview of the study selection process. A total of 8277 studies were identified by the searches after duplicate removal, 44 full-text articles were screened, 12 studies [24–35] met the inclusion criteria and eight provided data that were suitable for meta-analysis. The search returned one study [36] which presented follow-up data from two separate trials [25, 34] which were considered separately in the meta-analysis.

### Study characteristics

The characteristics of the included studies are detailed in Table 1 and are described briefly below. The 12 included studies were published from 2010 to 2018. The majority of studies were conducted in the USA (*N* = 6), followed by the United Kingdom (*N* = 3), Australia (*N* = 1), Finland (*N* = 1) and Spain (*N* = 1). Physical activity was objectively measured in all studies, using an accelerometer (9 studies), a step-activity monitor (1 study), or cardiorespiratory fitness testing (2 studies). Two studies had a mean participant age of 70 years or more, a further nine involved participants with a mean age from 50 to 69 years and one study involved younger adults with a mean age of 28 years. The interventions studied either promoted walking or general physical activity and were delivered at participants’ homes or in community centres. The control groups received either minimal physical activity promotion (e.g. an informational booklet), generic healthy living advice, or usual care.

**Table 1:** Summary of included studies characteristics

Study ID	Participant characteristics	Interventions	A. Physical Activity assessment method B. Monitoring duration C. Valid time criterion	Outcomes
**Cochrane 2017 RCT **[24]	1590 participants (790 intervention, 800 control), sedentary older adults with cardiovascular-related conditions and high risk of mobility disability, 67.2% women, Age mean = 79, multiple ethnicities, 23.6% ethnic minorities. Country: United State of America	**Intervention group** physical activity including walking towards 150 min/week, strength, flexibility and balance training **Control group** information on health education awareness of older adult health Duration: 6 months	A. Waist- accelerometer B. 7 days C. 10 h /day, 3 days of 7	Accelerometry: Step count, 30 min peak cadence, time at 100 to 499 counts/min, and ≥ 500 counts/min. 12 and 24 month follow-up
**Davies 2016** **RCT **[25]	Cluster randomised RCT (20 clusters/433 participants in the control group and 23 clusters/447 participants in the intervention) with pre-diabetes. Mean age: 64 years Country: United Kingdom	**Intervention group**: A group structured education programme with annual refresher and regular phone contact. **Control group**: Standard care Duration: 6 h intervention delivered in English, 12 h when intervention delivered in Guajarati	A. Sealed pedometer B. 7 days C. 10 h /day, 3 days of 7	Median daily step count at 6, 12, 24 and 36 months
**Eakin 2014 RCT **[26]	302 participants (151 intervention, 151 control) with type 2 diabetes with BMI^c^ 33.1(6.1). Mean age: 58 Country: Australia	**Intervention group** telephone delivery of combined approach on increasing PA, reduce energy intake and behaviour therapy with a workbook with intervention detail. **Control group** usual care education brochures on diabetes self-management Duration: 18 months	(A) Accelerometer (B) 7 days (C) 10 h/day, 4 days of 7	Accelerometry: Time at ≥ 574 counts/min and ≥ 2743 counts/min. 24 month follow-up.
**Fielding 2017 RCT **[27]	1635 participants (818 intervention, 817 control), sedentary older adults with high risk of mobility disability, 68.4% women, Age mean: 78, 69.2% White, multiple ethnicities. BMI^c^: 31.4(6.4). Country: United State of America	**Intervention group** structured moderate intensity aerobic walking 400 m in 15 min, resistance, flexibility and balance training; **Control group** age-specific health information in the community: home(unsupervised), centre (supervised) Duration: 6 months	(A) Waist- accelerometer (B) 7 days (C) (600 min)10 h/day, 5 days of 7	Accelerometry: Time at ≥ 760 counts/min. 12 and 24 month follow-up
**Grandes 2011 CRCT **[28]	3691 participants (1906 intervention, 1785 control), sedentary older adults’ patients with high risk of cardiovascular-related conditions, 64.6% women, Age mean = 51, VO2_max_ 24.4ml/kg/min Country: Spain	**Intervention group** 12wks PA prescription and online material to improve PA + 15 min of advice on PA plan **Control group** usual GP care with no specific physical activity advice unless patient’s health problems are directly related to inactivity Duration: 3 months	YMCA Cycle ergometer submaximal exercise test: Estimated maximum oxygen uptake (VO_2max_ ml/kg/min)	Cardiorespiratory fitness (VO_2max_ ml/kg/min). 6, 12 and 24 months follow-up
**Harris 2018 RCT **[29]	PACE-UP = 1,023 (Postal:339, Nurse: 346, control: 338); 64% women, age mean:59 PACE-Lift = 298 (Intervention: 150, control: 148), 53%women, age mean 67, sedentary older adults’ patients with high risk of mobility disability, multiple ethnicities Country: United Kingdom	**Intervention group** structured moderate intensity walking **Control group** usual GP practice Duration: 12 months	(A) Waist- accelerometer (B) 7 days (C) 9 h/day, 5 days of 7	Accelerometry : daily step counts, MVPA^a^ min/wk ≥ 10 min bouts, change in sedentary time. PACE-UP 12 months and 3 years follow-up; PACE-Lift 12 month and 4 years follow-up.
**Komulainen 2010 RCT **[30]	1335 participants for 6 intervention arms [(Aerobic: 220, age mean 67, BMI^c^: 28.3(4.5); Resistance:220, age mean 66, BMI^c^: 27.2(4.1); Diet: 224, age mean 67, BMI^c^:27.3(4.3); Aerobic and Diet: 224, age mean 66, BMI^c^: 28.0(4.8); Resistance and Diet: 221, age mean 66, BMI^c^:27.3(4.6)); Control:226,age mean 66, BMI^c^: 27.7(4.3)] older adults with risk of cognitive impairment. Country: Finland	**Intervention group** individual physical activity programme such as aerobic, strength training, dietary or combined programme **Control group** information on diet and physical activity in the community Duration: 6 months	Cycle ergometer Accelerometer Step Activity Monitor	Cardiorespiratory fitness (VO_2max_ ml/kg/min). 12 and 24 months follow-up
**Unick 2016 RCT **[31]	2400 participants (1199 intervention, 1201 control), sedentary Type2 Diabetes (obese and overweight), 15.2% with history of CVD, 55.8% women, Age mean = 59, multiple ethnicities, BMI^c^: [I: 36.1(6.0), C:36.1(5.7)] Country: United State of America	**Intervention group** Intensive Lifestyle Intervention including reducing calorie intake and structured physical activity MVPA^a^≥175 min/wk plus counselling sessions throughout the study time **Control group** information on dietary support education, physical activity without specific behaviour adoption strategies Duration: 12 months	(A) Waist- accelerometer (B) 7 days (C) 10 h/day, 4 days of 7	Accelerometry: Bout-related MVPA^a^ (min/wk). 4 years of follow-up
**Unick 2017 RCT **[32]	599 participants (2 intervention arms Small Change (SC): 200, Large Change (LC): 197, Control Self-Guided SG: 202), 78% women, Age mean = 28, BMI^c^: 25.4(2.6), multiple ethnicities, 73% white. Country: United State of America	**Intervention group** physical activity MVPA^a^ ≥150 min/wk SC, ≥ 250 min/wk LC, 10,000 steps/day; in addition, both groups attended weekly personal and group sessions for 8 weeks, then 2 monthly sessions plus online material each following year of study. **Control group** information on weight gain prevention in one group session and a brief overview of the intervention approach. Duration: 4 months	(A) Sensewear Armband (B) 7 days (C) ≥ 8 h/day, 4 days of 7	Accelerometry: Bout-related MVPA^a^ (min/wk) and daily steps. 12–24 months follow-up
**Varma 2016 RCT **[33]	123 participants (65 intervention, 58 Control), sedentary older adults with risk of cognitive and functional decline), Aged ≥ 60 years Country: United State of America	**Intervention group** walking activity **Control group** A retirement education programme with short-duration volunteering opportunities and put on a waiting list for the intervention.	Accelerometer Step Activity Monitor	Accelerometry: step/day. 12–24 months follow-up
**Wilson 2015 RCT **[35]	434 participants (2 intervention police patrolled walking programme^b^ and social marketing: 133, Police patrolled walking programme^b^: 164, Control: 137), sedentary Caucasian adults with no life-threatening illness),62% women, Age mean 51, BMI^c^: 30.26(SE 0.39) Country: United State of America	**Intervention group** Police patrolled walking programme^b^ and social marketing and Police patrolled walking programme^b^ **Control group** invited to health education events, two per year Duration: 12 months	Accelerometer MVPA^a^> 3METs ≥ 1075 counts/min	Accelerometry: MVPA^a^. 18 and 24 months follow-up
**Yates 2017 CRCT **[34]	808 participants (423 intervention, Control: 385), adults with a risk of Type2 Diabetes, 64% male, age mean 63, BMI^c^: 32.4(5.5) Country: United Kingdom	**Intervention group** walking and increased daily activity **Control group** booklet of health education on diabetes prevention and management using physical activity and lifestyle change. Duration: 12 months	(A) Waist- accelerometer (B) 7 days (C) 10 h/day, 4 days of 7	Accelerometry: MVPA^a^≥1952c/m, step/day. 24 and 36 months follow-up

^a^*MVPA* Moderate to Vigorous Physical Activity. ^b^Police Patrolled Walking Programme: Scheduled walks were patrolled by an off-duty police officer; ^c^*BMI* Body Mass Index

### Risk of Bias

 The Risk of Bias results are summarised in Table 2 for the eight studies included in the two meta-analyses. Across all domains there were potential concerns about risk of bias in six studies. In four studies [27, 31–33], the concern arose from a lack of information provided on deviations from /adherence to the intended intervention. Two studies [24, 29] also provided insufficient information on blinding of outcome assessors. Two studies [26, 34] were classified as having a low risk of bias in all domains assessed. The studies not included in the meta-analysis [25, 28, 30, 35] had moderate to high risk of bias.

**Table 2:**
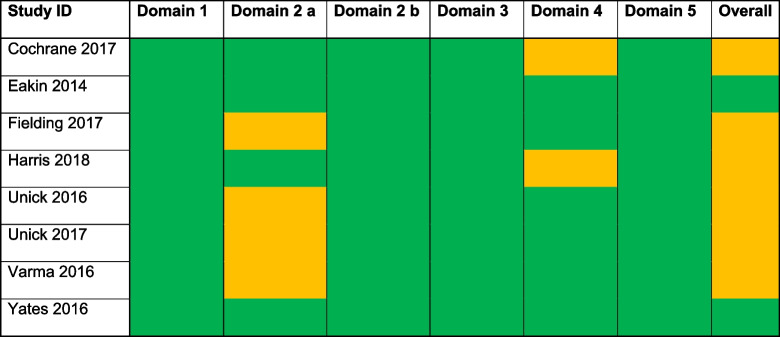
The risk of bias assessment for included studies

Green=Low, Amber=unclear. Red=high

### Synthesis of results

Of the twelve studies that met our inclusion criteria, four were not included in the meta-analyses, as they did not use comparable objective measures of physical activity or were cluster randomised control trials. These four studies are summarised as follows: Two [28, 30] used cardiorespiratory fitness ($\dot{V}$O_2peak_) as a surrogate measure for physical activity. One of these studies [30] randomised 1335 participants into six groups with a continuous (four year) intervention promoting combinations of diet, aerobic or resistance exercise. This showed no difference in $\dot{V}$O_2peak_, calculated by maximal exercise testing, between the control and any of the four exercise intervention arms; aerobic exercise, resistance exercise, aerobic exercise and diet, and resistance exercise and diet, after the first 2 years. A cluster randomised trial of exercise prescription in 4317 participants who did not meet minimal physical activity standards showed no significant difference in $\dot{V}$O_2max_ at 6, 12 and 24 months, when calculated by the YMCA cycle ergometer submaximal exercise test [37], between the control arm receiving normal care and the intervention arm [28]. A further three arm cluster randomised study [35] comparing increasing police foot patrols with marketing, without marketing and no intervention with the aim of increasing walking and physical activity in the African-America community found no significant difference across communities at 24 months for moderate-to-vigorous physical activity assessed using accelerometers. The final study [25] was also a cluster randomised control trial with 20 clusters (433 participants) in the intervention arm and 23 control clusters (447 participants), with the intervention arm receiving a six-hour education programme with annual refresher. A significant effect on step count was observed at 6, 12 and 36 but not 24 months. The remaining 8 studies, including 8480 participants, provided data that were suitable for meta-analysis.

### Physical activity outcomes

The Figures below (2, 3, 4 and 5) show the pooled and individual study results for physical activity measured in steps per day and minutes of moderate to vigorous physical activity per week at 24 months and beyond 24 months (range 36 to 48 months) of follow up.

**Fig. 2:**
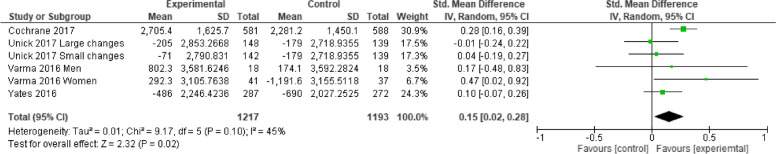
Meta-analysis of daily steps; control compared to intervention at 24 months

**Fig. 3:**
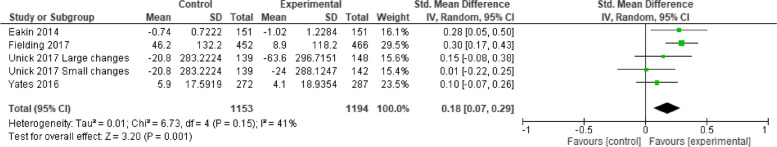
Meta-analysis of minutes per week of moderate to vigorous activity; control compared to intervention at 24 months follow up

**Fig. 4:**
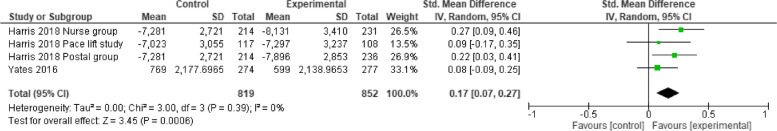
Meta-analysis of daily steps; control compared to intervention at 36–48 months

**Fig. 5:**
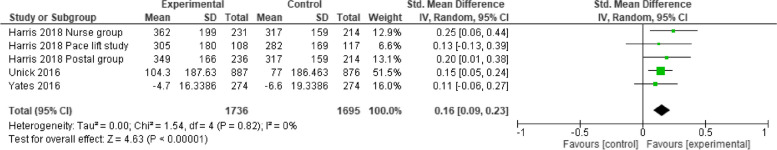
Meta-analysis of minutes per week of moderate to vigorous activity; control compared to intervention at 36–48 months

Figure 2 shows the meta-analysis for four trials (six intervention arms) with 2410 participants, illustrating the effects of physical activity intervention on daily steps at 24 months post baseline. The pooled results indicate a significant difference between intervention and control arms (SMD = 0.15, 95%CI; 0.02 to 0.28; I^2^ = 45%). Figure 3 shows the meta-analysis for four trials (five intervention arms) with 2,347 participants, illustrating the effects of physical activity intervention on moderate to vigorous physical activity (minutes per week) at 24 months post baseline (SMD = 0.18, 95%CI: 0.07 to 0.29; I^2^ = 41%).

Figure 4 shows the meta-analysis for two trials (four intervention arms) with 1671 participants, illustrating the effect of physical activity intervention on physical activity outcomes at 36 to 48 months (1672 participants, SMD = 0.17; 95%CI: 0.07 to 0.27; I^2^ = 0%). Figure 5 shows the meta-analysis for three trials (five intervention arms) with 3435 participants, illustrating the effect of physical activity interventions on moderate to vigorous physical activity at 36 to 48 months (SMD = 0.16; 95%CI: 0.09 to 0.23; I^2^ = 0%). All meta-analyses showed that the intervention group undertook significantly more physical activity than the control group.

There were two common research design components associated with statistical significance intervention success: large sample size (effective interventions at 36–48 months had 214 to 887 per group), and the use of accelerometery to measure physical activity. There were no intervention components that were clearly associated with success across the included studies.

## Discussion

This systematic review shows that physical activity interventions can deliver a small but significant increase in physical activity at 24 months and for up to 48 months of follow up. This should be interpreted with caution due to the relatively low effect sizes in all of the meta-analyses.

The low effect size may simply reflect the fact the standard deviations are generally high for measures of physical activity and it is more relevant to assess whether the effects observed are clinically meaningful. Assuming a standard deviation at 24 months of 118–140 min [34], the pooled mean SMD of 0.18 in our meta-analysis equates to a difference of 21–25 min per week of moderate to vigorous physical activity at 24 months, with the most effective intervention in our review delivering 35–42 min per week [29]. Researchers identify 30–60 min of moderate to vigorous physical activity as being a clinically meaningful change [5]. In terms of walking outcomes, assuming a standard deviation of 2123–8215 steps per day [34], the pooled mean SMD of 0.15 in our meta-analysis equates to 318–1232 steps per day, with the most effective intervention delivering 998–3861 steps per day. Previous research has identified 1000 steps per day as being clinically meaningful [5, 38]. However, it is important to note that evidence and clinical guidance now recognise that any increase in physical activity in previously inactive adults is important for health [5, 39, 40].

It is unclear what intervention components or behaviour change techniques were associated with significant long-term changes in physical activity. Previous research has shown that the behaviour change techniques “prompt self-monitoring of behavioural outcomes” and “use of follow up prompts” significantly predict the success rate of physical activity interventions at up to 15 months of follow-up in young and middle-aged adults [41]. It is unclear if this is replicable in studies over 24 months and with adults of all ages. However, the two studies [29, 31] that showed a significant difference at 36–48 months both used the aforementioned behaviour change techniques, suggesting that they may be associated with longer-term effectiveness.

The findings of this systematic review support the finding of similar reviews. Madigan et al.’s review of the effectiveness of interventions on device-measured physical activity [19] reported a significant difference in steps per day at follow up between the control and intervention group (MD: 94.46, 95% CI: 65.12, 123.79). However, they did not set a minimum follow up time with two studies only following up participants for three months. Therefore they concluded that physical activity is maintained for at least 3 months in successful programmes. Our findings are partially supported by the findings of Chaudhry et al. [18] who found that step counter (step monitoring) interventions significantly improved steps at 3–4 years (MD: 494 95% CI: 251, 738) but not at 2 years (MD: 66 95% CI: -92, 224).

### Strengths and limitations

This is the first systematic review of physical activity interventions to exclusively focus on objective measures of physical activity. Hence it is likely that the results are more representative of actual physical activity which has been undertaken than reviews which also include self-reported physical activity [15, 16]. The methodological quality of the included papers was moderate to high. However, there are several limitations that need to be acknowledged. The low number of included studies, along with heterogeneous interventions and populations and a lack of information on interventions in many cases [42] makes it difficult to identify intervention characteristics that were associated with effectiveness.

In addition, one study [37] required participants to complete a two week period of self-monitoring of physical activity and diet before randomisation which resulted in 116 potential participants dropping out of the study. It is unclear what affect this had on the study results however this may have artificially inflated intervention adherence and the intervention effect for this study. Despite this, any such effect is likely to be minimal as the study recruited 2400 participants in total.

Due to the cost of objective physical activity measurement, there may also be selection bias, as studies that are already established are more likely to receive funding for longer-term follow-up. Accelerometers and pedometers also do not accurately measure some activities e.g. cycling and swimming.

### Future research

Further high-quality research using objective measures is needed to identify the long-term effects of a wider range of interventions for promoting physical activity and to identify intervention content and delivery strategies that are associated with effectiveness.

In particular more research is needed to develop interventions with larger effect sizes, to identify effective lower intensity /lower cost approaches and to explore the cost-effectiveness of different approaches. The single existing example of an effective lower intensity intervention [29] needs to be replicated, perhaps in the context of an implementation trial [43]. The long-term effectiveness and cost-effectiveness of digital interventions to promote physical activity also needs to be assessed, as no studies of digital interventions with objective outcomes at 24 months or more were identified by our searches.

## Conclusion

This review shows that behavioural interventions can be effective for promoting objectively measured physical activity in the long-term. Although, the standardised mean differences observed were small, the changes in moderate-to-vigorous physical activity and steps were clinically meaningful in the better-performing interventions.

### Supplementary Information

** Additional file 1: Supplementary table 1.** Example search strategy for MEDLINE

## Data Availability

Available by contacting the corresponding author.
