# Supplementary material for: A systematic review and meta-analysis of the long-term effects of physical activity interventions on objectively measured outcomes
Source: BMC Public Health. 2023 Sep 2;23:1697. doi: 10.1186/s12889-023-16541-7 (PMC10474717; doi:10.1186/s12889-023-16541-7)
Supplement: Supplementary file 1 — Additional file 1: Supplementary table 1. Example search strategy for MEDLINE [file 12889_2023_16541_MOESM1_ESM.docx]

Supplementary table 1: Example search strategy for MEDLINE.

| ID | Search |
| --- | --- |
| #1 | physical activity:ti,ab |
| #2 | physical exertion:ti,ab |
| #3 | physical fitness:ti,ab |
| #4 | physical training:ti,ab |
| #5 | exercis*:ti,ab |
| #6 | sport*:ti,ab |
| #7 | Yoga:ti,ab |
| #8 | Pilates:ti,ab |
| #9 | tai chi:ti,ab |
| #10 | balance exercis*:ti,ab |
| #11 | dancing:ti,ab |
| #12 | walk*:ti,ab |
| #13 | bicycle*:ti,ab |
| #14 | bike*:ti,ab |
| #15 | cycling:ti,ab |
| #16 | swim*:ti,ab |
| #17 | aerobic exercises:ti,ab |
| #18 | Randomized Controlled trial*:ti,ab |
| #19 | Randomised Controlled trial*:ti,ab |
| #20 | crossover procedure*:ti,ab |
| #21 | single blind*:ti,ab |
| #22 | random allocation:ti,ab |
| #23 | cluster randomized:ti,ab |
| #24 | comparative study:ti,ab |
| #25 | objectiv* near/3 measur*:ti,ab |
| #26 | objectively measured:ti,ab |
| #27 | accelerometry:ti,ab |
| #28 | accelerometer*:ti,ab |
| #29 | Pedometer*:ti,ab |
| #30 | Pedometry:ti,ab |
| #31 | heart rate monitor:ti,ab |
| #32 | cardiorespiratory fitness:ti,ab |
| #33 | cardiopulmonary exercise test:ti,ab |
| #34 | cardiopulmonary fitness:ti,ab |
| #35 | CPEX:ti,ab |
| #36 | CPET:ti,ab |
| #37 | energy expenditure:ti,ab |
| #38 | Jawbone:ti,ab |
| #39 | Fitbit:ti,ab |
| #40 | Nike Fuelband:ti,ab |
| #41 | Samsung Gear fit:ti,ab |
| #42 | Microsoft band:ti,ab |
| #43 | new lifestyles:ti,ab |
| #44 | Lifecorder Ex:ti,ab |
| #45 | GENEActiv:ti,ab |
| #46 | Omron:ti,ab |
| #47 | activPal:ti,ab |
| #48 | tritrac:ti,ab |
| #49 | tracmor:ti,ab |
| #50 | Actigraph:ti,ab |
| #51 | Yamax:ti,ab |
| #52 | StepWatch:ti,ab |
| #53 | PolarS410:ti,ab |
| #54 | Actiheart:ti,ab |
| #55 | SenseWear:ti,ab |
| #56 | misfit:ti,ab |
| #57 | {OR #1-#17} |
| #58 | {OR #18-#24} |
| #59 | {OR #25-#56} |
| #60 | (#57 AND #58 AND #59) |
|  | Trials |
